# Disparities in quantification of mitral valve regurgitation between cardiovascular magnetic resonance imaging and trans-thoracic echocardiography: a systematic review

**DOI:** 10.1007/s10554-024-03280-y

**Published:** 2024-11-05

**Authors:** Sulayman el Mathari, Rahul A. Bhoera, Luuk H. G. A. Hopman, Josephine Heidendael, Arjan Malekzadeh, Aart Nederveen, Pim van Ooij, Marco J. W. Götte, Jolanda Kluin

**Affiliations:** 1 Department of Cardiothoracic Surgery, Amsterdam University Medical Center, Amsterdam, The Netherlands; 2 Department of Cardiology, Amsterdam University Medical Center, Room D3-221, Meibergdreef 9, 1105 AZ Amsterdam, The Netherlands; 3 Medical Library, Amsterdam University Medical Center, Amsterdam, The Netherlands; 4 Department of Radiology and Nuclear Medicine, Amsterdam University Medical Center, Amsterdam, The Netherlands; 5 Department of Cardiothoracic Surgery, Erasmus University Medical Center, Rotterdam, The Netherlands

**Keywords:** Mitral valve regurgitation, Transthoracic echocardiography, Magnetic resonance imaging

## Abstract

Primary mitral regurgitation (MR) is a prevalent valvular heart disease. Therapy stratification for MR depends on accurate assessment of MR severity and left ventricular (LV) dimensions. While trans-thoracic echocardiography (TTE) has been the standard/preferred assessment method, cardiovascular magnetic resonance imaging (CMR) has gained recognition for its superior assessment of LV dimensions and MR severity. Both imaging modalities have their own advantages and limitation for therapy guidance. However, the differences between the two modalities for assessing/grade severity and clinical impact of MR remains unclear. This systematic review aims to evaluate the differences between TTE and CMR in quantifying MR severity and LV dimensions, providing insights for optimal clinical management. A literature search was performed from inception up to March 21st 2023. This resulted in 2,728 articles. After screening, 22 articles were deemed eligible for inclusion in the meta-analysis. The included study variables were, mitral valve regurgitation volume (MR_VOL_), regurgitation fraction (MR_FRAC_), LV end-diastolic volume (LVEDV), LV end-systolic volume (LVESV), LV stroke volume (LVSV), and LV ejection fraction (LVEF). TTE showed a significant higher MR_VOL_ (10.4 ml, I^2^ = 88%, p = 0.002) and MR_FRAC_ (6.3%, I^2^ = 51%, p = 0.05) compared to CMR, while CMR demonstrated a higher LVEDV (21.9 ml, I^2^ = 66%, p =  < 0.001) and LVESV (16.8 ml, I^2^ = 0%, p =  < 0.001) compared to TTE. Our findings demonstrate substantial disparities in TTE and CMR derived measurements for parameters that play a pivotal role in the clinical stratification guidelines. This discrepancy prompts a critical question regarding the prognostic value of both imaging modalities, which warrants future research.

**Graphical abstract:**

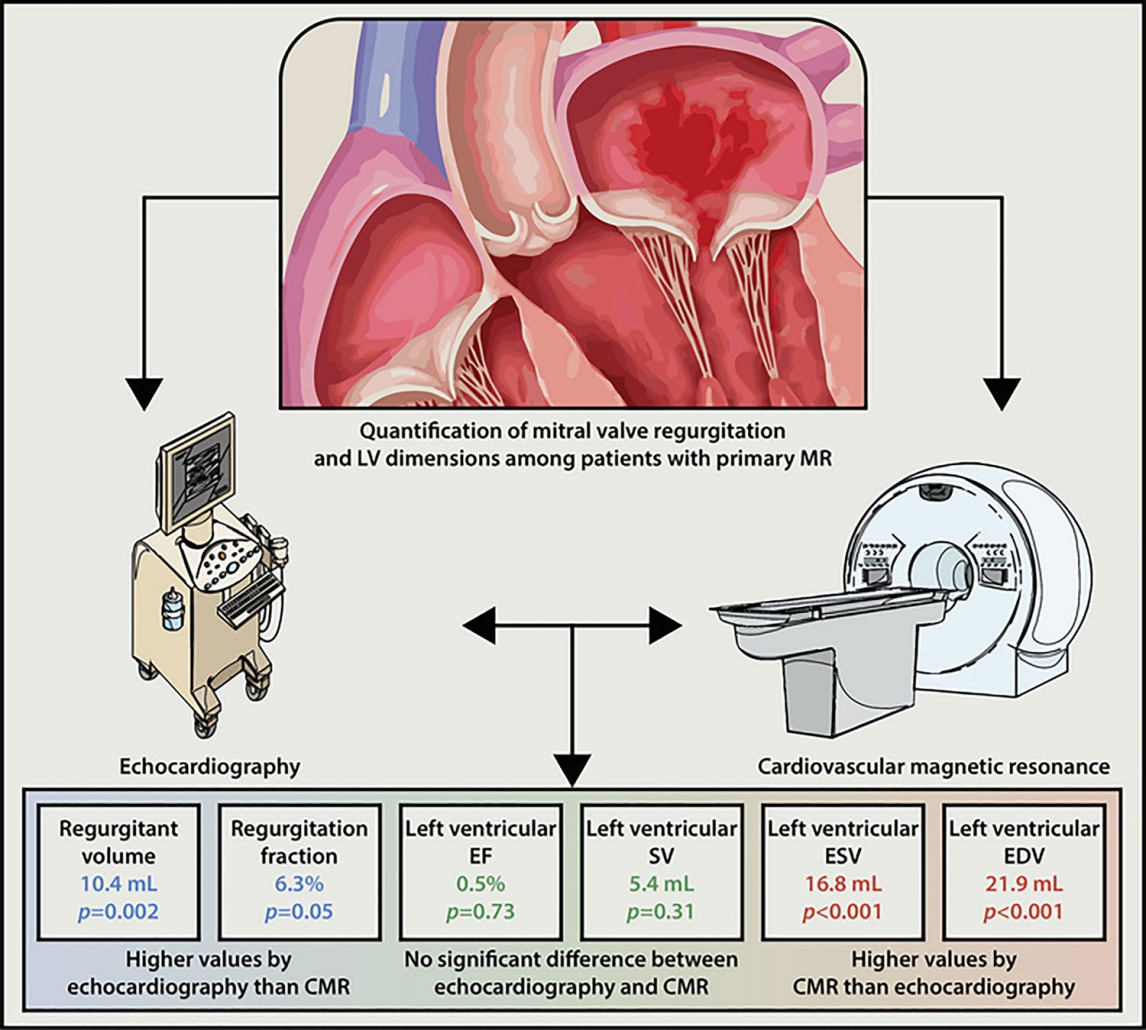

**Supplementary Information:**

The online version contains supplementary material available at 10.1007/s10554-024-03280-y.

## Introduction

Primary mitral valve regurgitation (MR) is a common valvular heart disease, with a prevalence of 2.3% in adults above the age of 60 [1, 2]. It is characterized by mitral valve (MV) dysfunction, resulting in backward flow of blood from the left ventricle (LV) into the left atrium (LA). Patients with severe MR may remain stable over a long period, but can deteriorate in a short period. [3]. Symptoms of dyspnea, edema and arrhythmias may appear [4], which are indicative of associated adverse events including heart failure, pulmonary arterial hypertension, atrial fibrillation and increased mortality [5, 6]. Therefore, it is crucial to diagnose and manage primary MR in an early phase, to prevent progression of disease, to anticipate complications and to improve patient outcomes.

Within the management of MR, there are two main challenges for optimal patient management. These are (1) accurate grading of MR severity, and (2) timely recognition of cardiac remodeling. Gold standard therapy for severe MR is MV repair surgery [7, 8]. Surgery is indicated when left ventricular ejection fraction (LVEF) is ≤ 60%, left ventricular end systolic diameter (LVESD) is ≥ 40 mm, and when there is an expectation that the surgical outcome will be durable [7]. This requires adequate monitoring to detect changes in MR severity and LV parameters for optimal timing for surgical intervention, especially in asymptomatic patients [9–11].

Clinicians rely on the quantification of MR severity and LV dimensions using appropriate imaging modalities [12]. Transthoracic echocardiography (TTE) is the gold standard for diagnosis and grading of MR, with established prognostic predictive value [13]. Particularly, TTE has been widely used due to its versatility and cost-effectiveness [14]. However, in addition to its operator-dependent nature, TTE also has intrinsic limitations, including a small field of view and the lack of tissue characterization capabilities. [12, 15]. Most importantly, the assessment of volumes, such as MR volume (MR_VOL_) and LV volumes, is based on calculations and assumptions that are prone to errors [16].

Cardiovascular magnetic resonance imaging (CMR) may serve as an alternative to monitor MR patients, due to its advanced imaging capabilities. CMR has proven effective in quantifying MV disease, particularly in assessing LV dimensions and hemodynamic consequences of severe MR [17–19]. Furthermore, CMR-derived results are more reproducible compared to TTE [20].

Previous studies suggest a discordance between the assessment of MV disease between TTE and CMR [12, 17, 19, 21, 22]. These studies, however, are small and demonstrate variable results in which both MR quantification and LV dimensions seem to differ in measured values between the two imaging modalities [23]. This divergence underscores the importance of ensuring accurate and consistent measurements, as it directly affects clinical therapy stratification.

Therefore, the aim of this review is to evaluate the discrepancies between TTE and CMR in quantifying essential parameters to determine MR severity and LV dimensions for optimal treatment stratification in patients with primary MR. For this purpose, we conducted a systematic review and meta-analysis. By critically appraising the available evidence, this study aims to provide valuable insights into the comparative accuracy and reliability of these imaging modalities for optimal clinical assessment and management in patients with primary MR.

## Methods

The conduct and reporting of this systematic review was in accordance with the Preferred Reporting Items for Systematic Reviews and Meta-Analyses (PRISMA)-statement [24] and registered in PROSPERO (registration number CRD42023446548).

### Search strategy

A comprehensive search was performed in the databases: Medline(Ovid), Embase(Ovid), CINAHL(Ebsco), Web of science, Scopus and CENTRAL (Cochrane library) from January 1st 2000 up to March 21st 2023, in collaboration with a medical information specialist (AM). The search included controlled terms and free text terms for synonyms of ‘mitral regurgitation’ combined with synonyms of ‘TTE’ and ‘cardiovascular magnetic resonance’. The search was performed without restrictions for date or languages. A search filter was applied to exclude animal studies, case reports, conference abstracts and letters. The full search strategies can be found in appendix A. Duplicate articles were excluded by an in-house made deduplication tool.

### Selection process

Studies were included if they met the following criteria: (1) comparison of primary MR quantification between TTE (2D and 3D) versus CMR with; (2) usage of the proximal iso-velocity surface area (PISA) for TTE [25]; and (3) the standard method for CMR by subtracting aortic phase contrast (AoPC) derived forward volume from LVSV [26]. Eligible studies must provide data for at least one of the following parameters: MR_VOL_ (mL), MV regurgitation fraction (MR_FRAC_ (%)), a representation of MR_VOL_ as a proportion of the LV stroke volume (LVSV (mL)) [27], LV end-diastolic volume (LVEDV (mL)), LV end-systolic volume (LVESV (mL)), and LV ejection fraction (LVEF (%)), since these parameters are relevant for clinical decision making in patients with MR [7].

Methodologically, data should be reported with mean differences and standard errors (SE) for the parameters studied. Studies were excluded if they met the following criteria: use of transesophageal TTE (TEE) or use of 4D flow CMR. Two reviewers (S.e.M. and R.B.), independently screened potentially relevant titles and abstracts for eligibility. If necessary, the full text article was checked for the eligibility criteria. Differences were resolved through a consensus procedure.

### Data extraction

Mean and SE values for MR_VOL_, MR_FRAC_, LVEDV, LVESV, LVSV and LVEF, together with the number of included patients, were extracted from all available studies. The severity of MR (mild, mild-moderate, moderate, moderate-severe, or severe) was used to characterize the different study populations. In addition, the agreement between TTE and CMR was also obtained for each study.

### Statistics

The mean difference between TTE and CMR was used for the meta-analysis. A random effect model together with pooled proportions of the studies was used to calculate 95% confidence intervals (95% CI). For each variable studied to classify MR (MR_VOL_, MR_FRAC_) and to assess global LV dimensions and function (LVEDV, LVESV, LVSV and LVEF), forest plots were generated to determine the differences between studies, to evaluate over- or underestimations of one method versus the other, and to assess the degree of heterogeneity between the results. Heterogeneity (I^2^) was assessed in the meta-analysis, with I^2^ > 50% as threshold for substantial heterogeneity, and a p-value ≤ 0.05 was considered to be statistically significant [28]. Meta-analyses and pooling were performed with *Review Manager version 5.4* (Copenhagen: The Nordic Cochrane Center, The Cochrane Collaboration, 2014).

## Results

### Search results

**Fig. 1:**
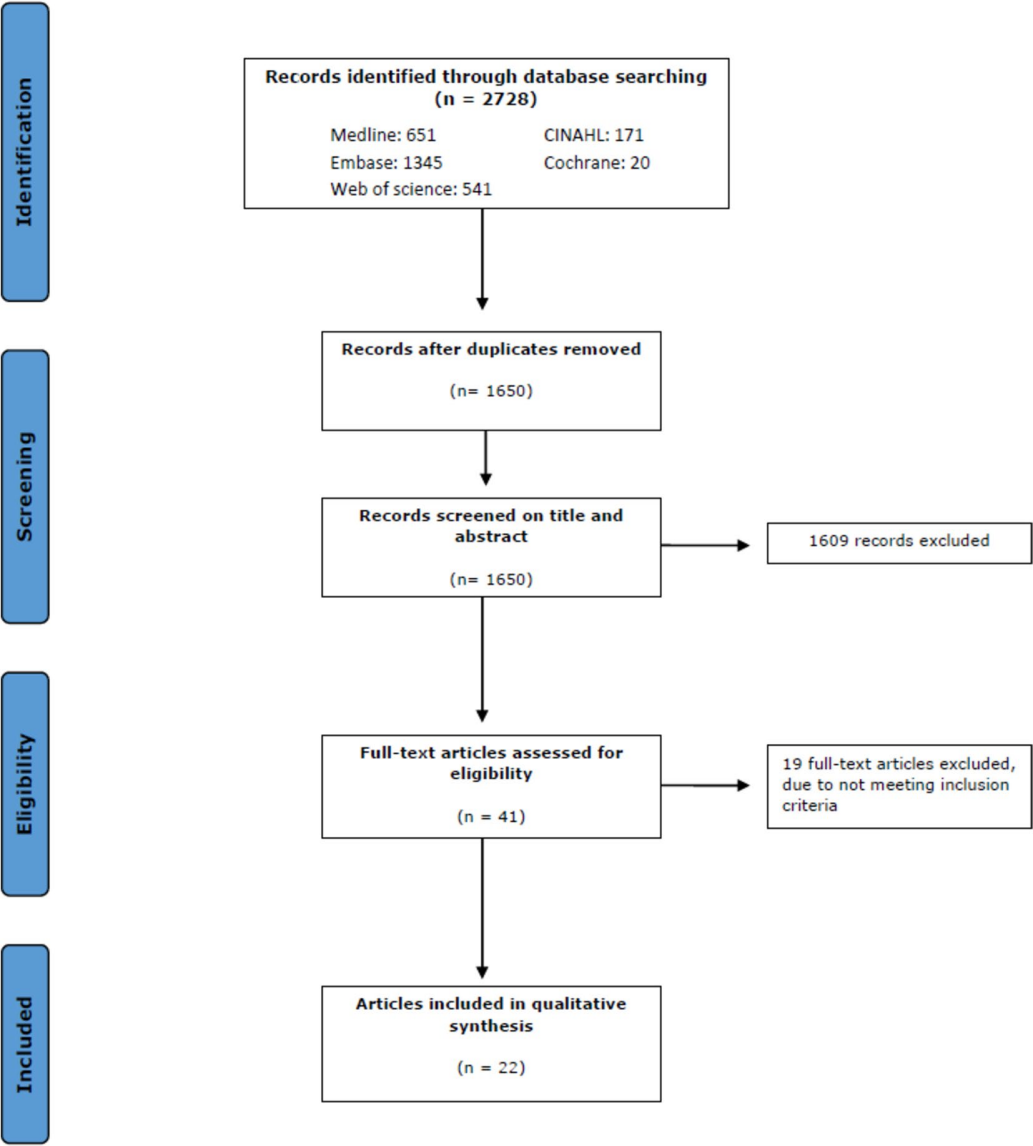
Flowchart of the search and selection procedure of studies

Results of the search are summarized in Fig. 1 and Table 1. The literature search generated a total of 2,728 references; 651 in Medline, 1345 in Embase, 541 in Web of Science, 171 in CINAHL, 20 in CENTRAL. After removing duplicate references, a total of 1,650 unique references remained for further evaluation. After title and abstract screening, 41 references remained for full text screening. Among these, 19 references did not meet the predetermined in- and exclusion criteria and were excluded from the study. Finally, 22 references were deemed eligible and included in the meta-analyses.

**Table 1:** Characteristics of included studies

Study	Year	Included patients TTE (n)	Included patients CMR (n)	Gender (male %)	Age (Mean ± SD)	Variables included for TTE and CMR	Severity of mitral regurgitation	Agreement between TTE and CMR (+ good agreement, – poor agreement ± moderate agreement)
Brugger et al. [29]	2015	60	60	65	67 ± 16	MR_VOL_, LVSV	Mild, moderate and severe	+
Capron et al. [30]	2020	44	44	75	60 ± 13	RV, LVEDV, LVESV, LVEF	Severe	No agreement reported
Cawley et al. [31]	2013	26	26	57.8	54 ± 13	MR_VOL_	Mild, moderate and severe	No agreement reported
Choi et al. [32]	2014	211	52	52.6	58.6 ± 14.9	MR_VOL_, LVEF	Mild, moderate and severe	No agreement reported
El Tahlawy et al. [33]	2021	30	30	50	52.7 ± 19.3	MR_VOL_, LVEDV, LVESV, LVEF	Mild, moderate and severe	±
Guo et al. [34]	2009	51	51	43.1	40 ± 10	LVEDV, LVESV, LVEF, LVSV	Mild, moderate and severe	±
Hamada et al. [35]	2012	46	46	39.5	71 ± 13	MR_VOL_	Mild, moderate and severe	No agreement reported
Hassan et al. [36]	2020	50	50	46	47.1 ± 16.8	MR_VOL_, LVEDV	Mild, moderate and severe	–
Heo et al. [37]	2017	101	27	51.3	57.4 ± 14.8	MR_VOL_	–	+
Jang et al. [38]	2018	33	33	81.8	52 ± 9	MR_VOL_	–	±
Le Goffic et al. [39]	2015	34	34	70.5	51 ± 19	MR_FRAC_, LVEDV, LVESV	Mild, moderate and severe	+
Levy et al. [40]	2018	53	53	69.8	64 ± 12	MR_VOL_, LVEDV, LVESV, LVEF, LVSV	Mild, moderate and severe	+
Levy et al. [41]	2021	157	157	71.3	62 ± 15	MR_VOL_	Mild, moderate and severe	No agreement reported
Lopez-Mattei et al. [42]	2016	60	60	60	61 ± 16	MR_VOL_, MR_FRAC_, LVEDV, LVESV	Mild, moderate and severe	No agreement reported
Militaru et al. [43]	2020	51	51	70.5	63 ± 16	MR_VOL_, MR_FRAC_, LVEDV, LVESV	Mild, moderate and severe	±
Penicka et al. [12]	2018	62	62	64.5	64 ± 12	MR_VOL_, LVEF	Severe	±
Shanks et al. [44]	2010	30	30	33.3	63.3 ± 11.6	MR_VOL_	Moderate and severe	+
Son et al. [45]	2013	30	30	50	56 ± 13	MR_VOL_	Mild, moderate and severe	±
Spampinato et al. [46]	2021	54	54	77.8	57 ± 14	MR_VOL_, MR_FRAC_, LVEDV, LVESV, LVEF, LVSV	Mild, moderate and severe	+
Uretsky et al. [47]	2022	73	73	63	61 ± 13	MR_VOL_	Mild, moderate and severe	+
Uretsky et al. [48]	2018	112	112	56.3	63 ± 14	MR_VOL_	–	±
Van de Heyning et al. [49]	2013	38	38	78.9	57 ± 14	MR_VOL_, LVEDV, LVESV, LVEF	Moderate and severe	+

*CMR* cardiac magnetic resonance, *LVEF* left ventricular ejection fraction, *LVEDV* left ventricular end-diastolic volume, *LVESV* left ventricular end-systolic volume, *LVSV* left ventricular stroke volume, *MR*_*FRAC*_ mitral regurgitation fraction, *MR*_*VOL*_ mitral regurgitation volume, *TTE* transthoracic echocardiography

### Study and patient characteristics

In total, 22 articles were included with a pooled number of 1,508 patients (mean age 58.3 ± 14 years and 60.4% was male). Table 1 displays key features and general outcomes of each study. MR_VOL_ was reported in 20/22 studies, MR_FRAC_ in 4/22 and LVEF in 7/22. LVEDV was reported in 10/22 studies, LVESV in 9/22 and LVSV in 4/22 articles. Six studies did not report overall agreement between TTE and CMR [30–32, 35, 41, 42]. Details about the grading of severity of MR were missing in three studies [37, 38, 48]. Furthermore, only one article assessed all variables of interest for this review [46]. The included studies cover a period from 2009 [34] to 2022 [48].

### Critical appraisal

To determine risk of bias, all included studies were assessed according to the QUIDAS-2 checklist [50]. Four domains were scored for each study; (1) patient selection; (2) index test (CMR); (3) reference standard (TTE), and (4) flow and timing.

**Fig. 2:**
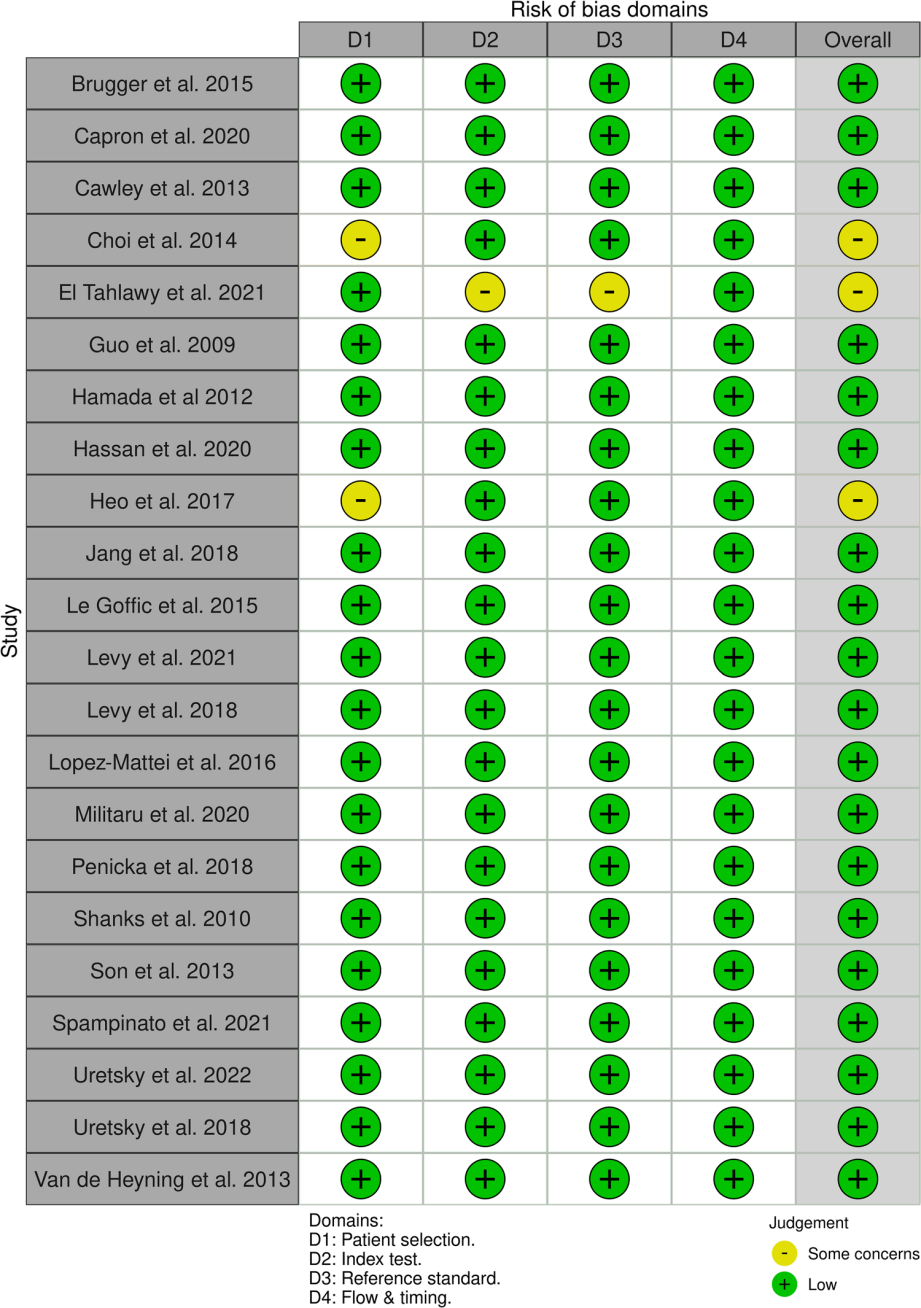
Critical appraisal according to the QUIDAS-2 checklist

Results are shown in Fig. 2. An overall low risk of bias was observed for all studies. Three studies were considered to have some concerns; in 2 studies only a subset of the total population underwent both TTE and CMR [32, 37] and 1 study did not report information about blinding [33]. However, these studies were still included in the meta-analysis because of a low risk on the other QUADAS-2 domains.

### Meta-analyses

**Fig. 3:**
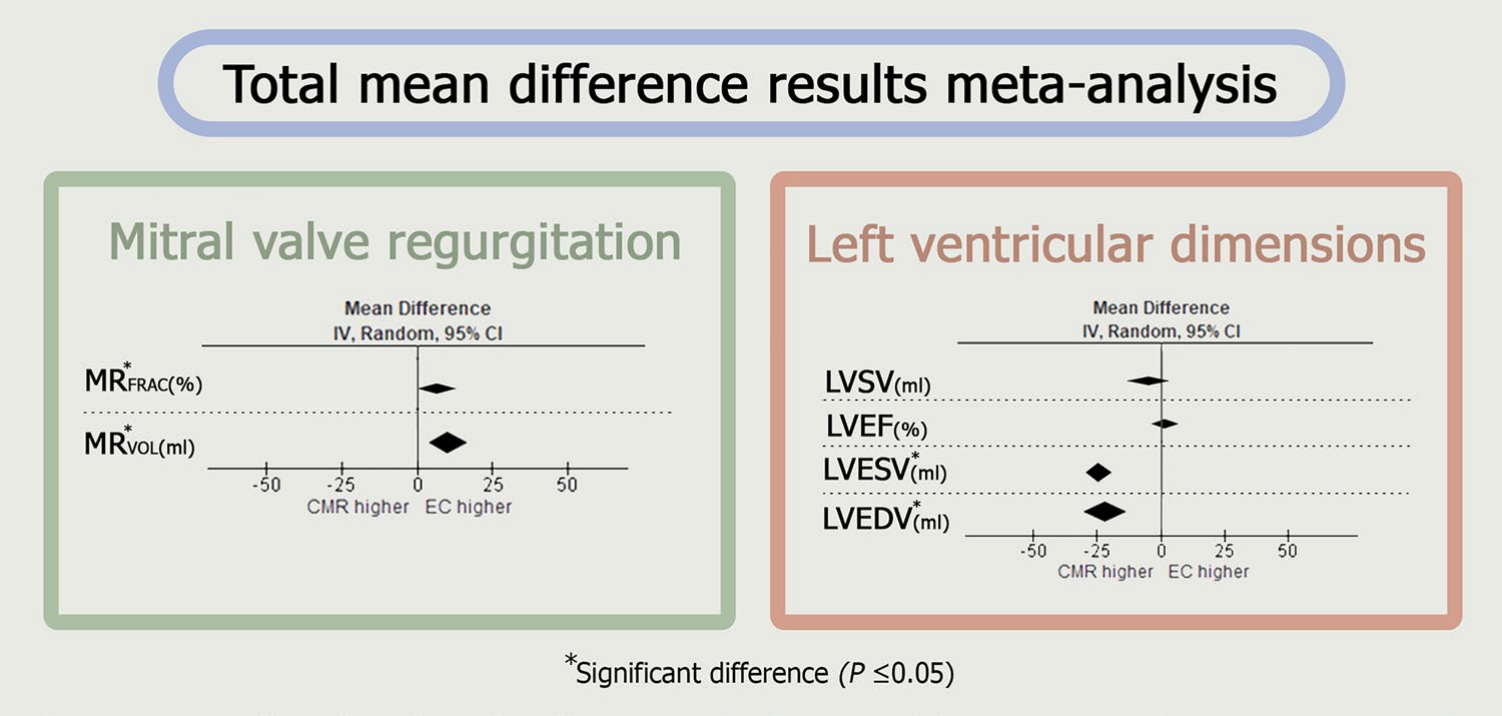
Forest plots of total mean difference results of all measured parameters divided in mitral valve regurgitation and left ventricular dimension parameters. *LVEF* left ventricular ejection fraction, *LVEDV* left ventricular end-diastolic volume, *LVESV* left ventricular end-systolic volume, *LVSV* left ventricular stroke volume, *MR*_*FRAC*_ mitral regurgitation fraction, *MR*_*VOL*_ mitral regurgitation volume

The total mean outcomes of the meta-analyses for all variables are presented in Fig. 3 and individual analyses for each parameter can be found in Supplemental 1, 2, 3, 4, 5, 6. The analysis revealed varying results for the different variables, with TTE suggesting a more severe degree of MR, and CMR indicating more severe LV remodeling.

**Fig. 4:**
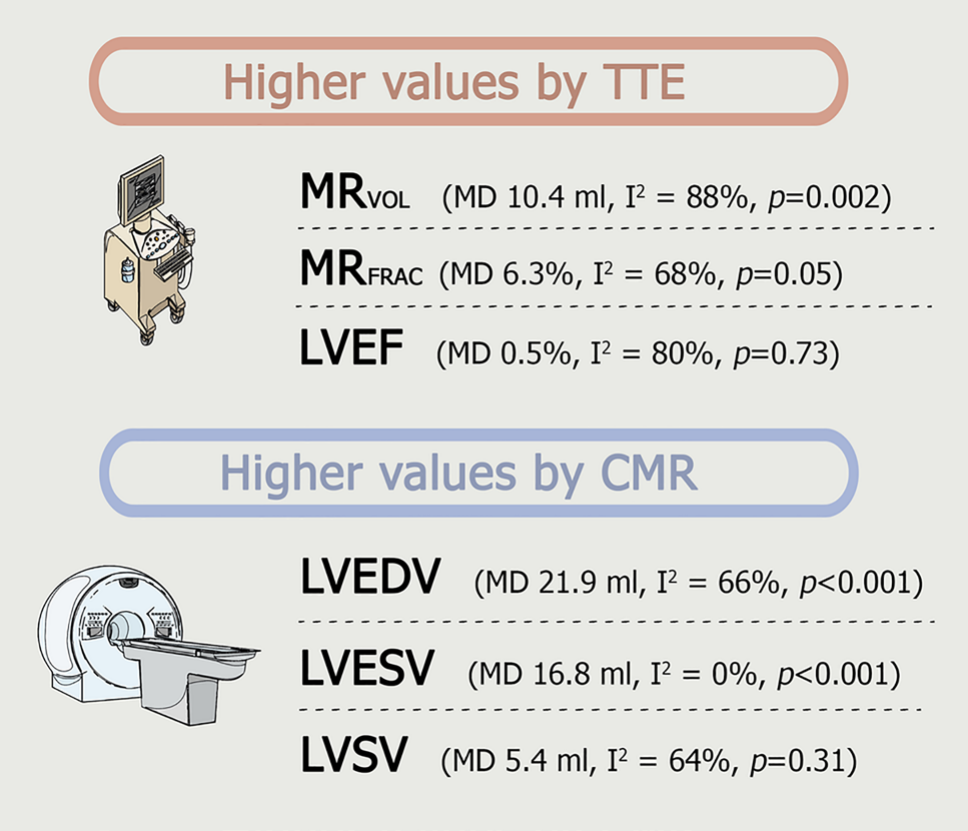
Summary of the meta-analyses mean difference (MD) results. Heterogeneity (I^2^) ≥ 50% is considered high. p-value ≤ 0.05 marks a significant difference between the included studies per variable. *CMR* cardiac magnetic resonance, *LVEF* left ventricular ejection fraction, *LVEDV* left ventricular end-diastolic volume, *LVESV* left ventricular end-systolic volume, *LVSV* left ventricular stroke volume, *MR*_*FRAC*_ mitral regurgitation fraction, *MR*_*VOL*_ mitral regurgitation volume, *TTE* transthoracic echocardiography

TTE showed a higher MR_VOL_ (mean difference 10.4 ml, I^2^ = 88%, p = 0.002 and higher MR_FRAC_ (mean difference 6.3%, I^2^ = 68%, p = 0.05) compared to CMR. However, CMR showed higher values compared to TTE for LV volumetric assessment; LVEDV (mean difference 21.9 ml, I^2^ = 66%, p < 0.001) and LVESV (mean difference 16.8 ml, I^2^ = 0%, p < 0.001). There was no significant difference for LVSV (mean difference 5.4 ml, I^2^ = 64%, p = 0.31) and LVEF (mean difference 0.5%, I^2^ = 80%, p = 0.73) between both imaging methods. The results of the meta-analyses are summarized in Fig. 4.

It is important to note that considerable heterogeneity (I2) was observed for each variable, indicating significant variations in the findings across the studies included in the meta-analyses. LVESV (I2 = 0%) was the only variable that exhibited a heterogeneity of less than 50%%, indicating a high level of homogeneity among the included studies for this variable. The other variables all show high heterogeneity (MRVOL [I2 = 88%], MRFRAC, [I2 = 68%], LVEDV [I2 = 66%], LVSV [I2 = 64%] and LVEF [I2 = 80%]. The results of the extensive heterogeneity analysis are summarized in Table 2.

**Table 2:** Results of heterogeneity analyses

Variable	Heterogeneity (I^2^)	p-value
MR_VOL_	**82%**	**0.002**
MR_FRAC_	**68%**	**0.05**
LVEDV	**66%**	** < 0.001**
LVESV	**0%**	** < 0.001**
LVSV	**64%**	**0.31**
LVEF	**80%**	**0.73**

Overall heterogeneity (I^2^) with corresponding p-value for each variable

*MR*_*VOL*_ mitral regurgitation volume, *MR*_*FRAC*_ mitral regurgitation fraction, *LVEF* left ventricular ejection fraction, *LVEDV* left ventricular end-diastolic volume, *LVESV* left ventricular end-systolic volume, *LVSV* left ventricular stroke volume

P-value <0.05 is considered significant

## Discussion

This systematic review with meta-analysis compared TTE and CMR in quantifying (severity of) MV disease in patients with primary MR. Assessed variables were MR_VOL_, MR_FRAC_, LVEDV, LVESV, LVSV and LVEF. TTE showed a significant higher MR_VOL_ (10.4 ml) and MR_FRAC_ (6.32%) compared to CMR, while CMR demonstrated a significantly higher LVEDV (21.8 ml) and LVESV (16.7 ml). These findings confirm the results of previous studies [12, 22, 23].

The existing Class Ia recommendations for MV repair surgery among patients with MR, include the classification of MR severity, LVEF ≤ 60% and LVESD ≥ 40 mm [7]. Therefore, it is of great clinical importance that our results illustrate the notable differences between the assessment of both MR severity grade (MR_VOL_ and MR_FRAC_) and LVESD when comparing measurements obtained by TTE and CMR. The established validated cut-off values for surgical intervention (i.e. MV repair) are based on studies using TTE. In current clinical practice, TTE is regarded the reference standard for diagnosing and stratifying patients suffering from MR [7]. This is mainly due to the practical use and cost-effectiveness of this easily accessible imaging modality. Nevertheless, because TTE tends to assess a higher MR_VOL_ but a lower LVEDV in comparison to CMR, this discrepancy could potentially lead to inconsistencies and discrepancies when determining the optimal timing for MV repair surgery, especially when using the echocardiographic cut-off values for CMR assessment.

The observed diagnostic differences between TTE and CMR can be explained by the fact that CMR is a volumetric technique with a high spatial resolution and a high contrast between blood pool and myocardial structures [51]. Nonetheless, CMR possesses limitations related to temporal resolution. In addition, the phase contrast method to quantify volumes and valvular function has some inherent limitations [52–54]. Therefore, if an assessment aims for precise global LV measurements, CMR could be the preferable choice. However, if the mechanism and severity of MR are studied, TTE is more beneficial due to higher clinically validated accuracy [55].

The results show high heterogeneity for MR_VOL_ (I2 = 88%), MR_FRAC_, (I2 = 68%), LVEDV (I2 = 66%), LVSV (I2 = 64%) and LVEF (I2 = 80%). This indicates high variability in the results of the included studies. Table 1 shows that not all studies included the same MR grades. Most studies included all grades (mild, moderate and severe); however, the results were not stratified by severity. Instead, mean values were reported in te results for the variables we utilized for the meta-analyses. This could potentially have caused the increased variability between the studies and thus the overall heterogeneity, since all patients in the included studies were assessed similarly utilizing the PISA method (TTE) and the volumetric method (CMR). Stratification by MR grade in future studies will enable more adequate analyses to assess heterogeneity for each variable.

It is crucial to emphasize that interpreting CMR results in patients with MR should not strictly adhere to current guideline recommendations, because these have been established based on values validated in studies using TTE [7]. Findings from previous studies that evaluated CMR threshold values associated with adverse prognostic outcomes in patients with MR have shown different cut-off values for MR_VOL_ ranging from 30 to 55 mL [11, 19, 26]. This relatively wide range underscores the current absence of a universally validated CMR-specific threshold value for severity of MR. For this reason, future studies should focus on evaluating the prognostic significance of different CMR cut-off values for MR severity based on MR_VOL_ and MR_FRAC_. Additionally, prospective research could explore the prognostic value of CMR-derived LVEDV and LVESV. This may lead to the establishment of distinct guideline cut-off values for both TTE and CMR, ensuring the optimal treatment for this specific patient population.

CMR’s superior contrast and enhanced spatial resolution, coupled with potential future technical advancements including 4D flow measurements, could position this advanced imaging modality as the future gold standard in MR severity assessment, as it already is for LV volumetric assessment [56, 57]. Meanwhile, TTE will remain an important modality to assess the mechanism of the MR because of higher temporal resolution. The two modalities are complementary to each other, supporting the importance of multimodality cardiovascular imaging.

It would have been preferable to also investigate potential disparities between TTE and CMR by including LA volume index (LAVi) in this meta-analysis, however only a limited number of small studies have compared LAVi between TTE and CMR [12, 30, 41]. LAVi gained a significant clinical relevance for MR patients since the introduction of the most recent ESC guidelines on valvular heart disease [7]. LAVi offers valuable insights into the extent of LA enlargement due to the increased strain resulting from the regurgitant flow of blood into the LA [58, 59]. Including this variable in a future meta-analysis would enhance our understanding of its comparative utility in the assessment of MR patients.

Lastly, it is important to recognize that MR severity and its clinical implications are multifactorial and should not be solely based on imaging parameters. A comprehensive evaluation must include the assessment of symptoms such as dyspnea, fatigue, and palpitations, as these provide valuable insights into the clinical impact of MR. The severity of symptoms plays a significant role in understanding the functional consequences of MR. Additionally, assessing a patient’s physical resilience, including their functional capacity and exercise tolerance, is essential for a complete clinical picture. Biomarkers, such as N-terminal pro B-type natriuretic peptide (NT-proBNP), are also integral to the assessment. NT-proBNP levels have been shown to correlate with MR severity and are associated with poorer outcomes. Therefore, they are important in guiding clinical decisions, particularly regarding the timing of surgical interventions for MR patients. Altogether, while advanced imaging techniques provide critical information for the quantification of MR, incorporating a holistic clinical assessment remains indispensable.

### Limitations

A major limitation was the presence of significant overall heterogeneity across all variables analyzed. The results show high heterogeneity for MR_VOL_ (I^2^ = 88%), MR_FRAC_, (I^2^ = 68%), LVEDV (I^2^ = 66%), LVSV (I^2^ = 64%) and LVEF (I^2^ = 80%). This indicates high variability in the results of the included studies. Table 1 shows that there is a variation in included MR severities among the studies. Most studies included all MR grades (mild, moderate and severe). Only four studies [34, 46, 60, 61] in our meta-analyses categorized participants based on MR severity. However, the results were not stratified by severity. Instead, only overall mean values were reported in the included studies which were used in this meta-analyses. This could have caused the increased variability and thus the overall heterogeneity. Moreover, also the relatively small cohort sizes of the included studies could have introduced sampling bias, impacting the robustness of our findings.

Since all patients in the included studies were assessed similarly utilizing the PISA method by TTE and the volumetric method by CMR, the calculation method for MR volume and fraction could not be a source of the observed heterogeneity. Analyses of differences between publication years, field of strength of CMR machines and tracing methods did not reveal a source heterogeneity.

Another noteworthy limitation is the exclusion of transesophageal TTE (TEE) from the screening process. This is because most existing studies investigating the discordance in MR quantification between TTE and CMR have primarily utilized TTE. However, it is important to acknowledge that TEE has demonstrated superiority over TTE in MR assessment [60, 61].

## Conclusion

A significant difference exists in the measurements of MR severity and LV volumes. TTE consistently shows higher values for MR_VOL_ and MR_FRAC_ compared to CMR, while CMR consistently shows larger values for LVEDV and LVESV. Given the essential role of both MR severity and LV volumes in clinical management for MR patients, the observed disparities in measurements could have notable clinical implications, particularly concerning decisions related to surgical timing. s prompts a critical question regarding the prognostic value of both imaging modalities, which warrants exploration in future research.

At present, TTE is the gold standard for diagnosing and clinically stratifying patients with MR. However, TTE suffers from inherent limitations that could have potential implications for the use in this context. CMR may overcome these limitations and play a significant role in the future of diagnosing, monitoring and stratifying MR patients, but requires adjusted cut-off values for clinical decision making.

## Supplementary Information

Below is the link to the electronic supplementary material.

Supplementary file1 (DOCX 92 KB)

## Data Availability

All data including the analysis are available upon request.
