# Supplementary material for: Disparities in quantification of mitral valve regurgitation between cardiovascular magnetic resonance imaging and trans-thoracic echocardiography: a systematic review
Source: Int J Cardiovasc Imaging. 2024 Nov 5;41(4):647–58. doi: 10.1007/s10554-024-03280-y (PMC11982156; doi:10.1007/s10554-024-03280-y)
Supplement: Supplementary file 1 — Supplementary file1 (DOCX 92 KB) [file 10554_2024_3280_MOESM1_ESM.docx]

**Supplement files**


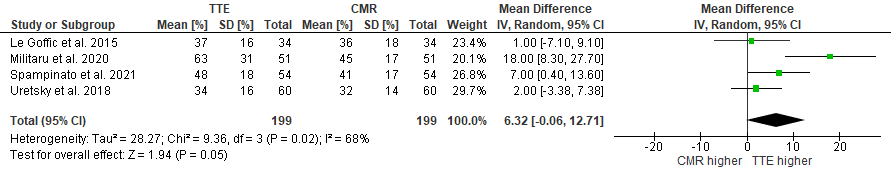
 **Supplemental 1.** Mitral valve regurgitation fraction. Forest plot of meta-analyses comparing TTE and CMR for MR_FRAC_


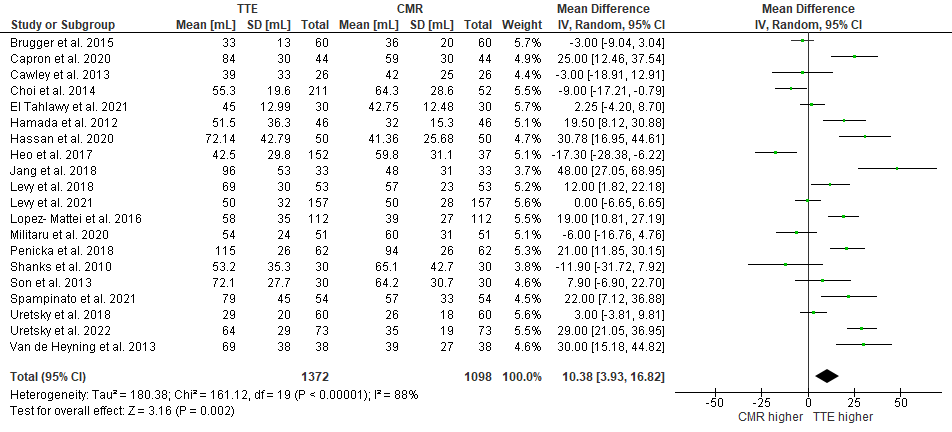


**Supplemental 2**. Mitral valve regurgitation volume. Forest plot of meta-analyses comparing TTE and CMR for MR_VOL_.


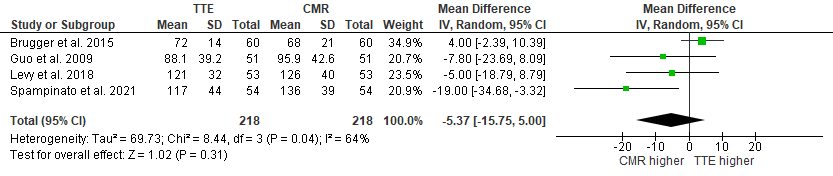
 **Supplemental 3.** Left ventricular stroke volume. Forest plot of meta-analyses comparing TTE and CMR for LVSV.


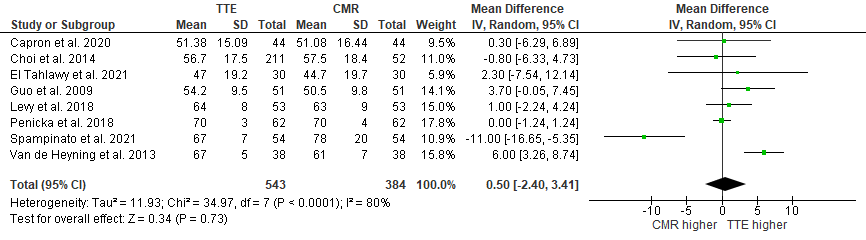
 **Supplemental 4.** LVEF Forest plot of meta-analyses comparing TTE and CMR for LVEF.


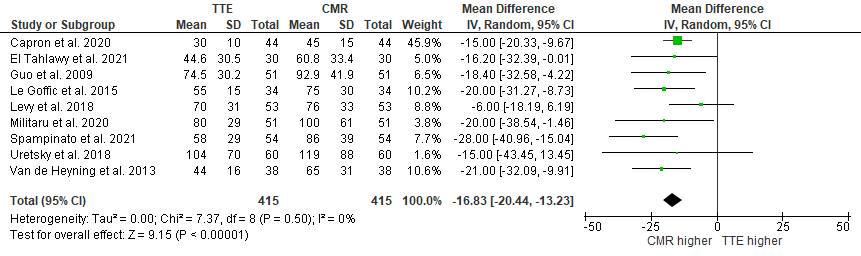


**Supplemental 5.** Left ventricular end-systolic volume. Forest plot of meta-analyses comparing TTE and CMR for LVESV.


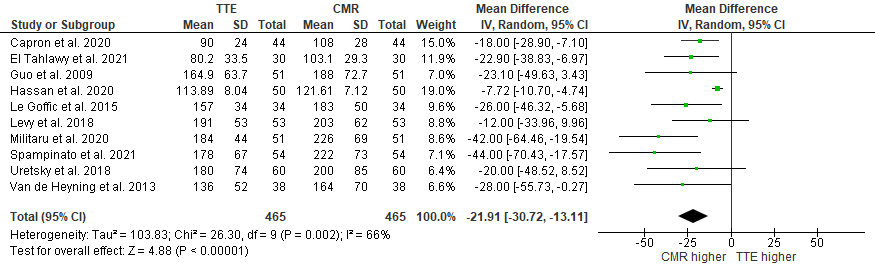


**Supplemental 6.** Left ventricular end-diastolic volume. Forest plot of meta-analyses comparing TTE and CMR for LVEDV.
